# Biodegradable Polyphosphazene Based Peptide-Polymer Hybrids

**DOI:** 10.3390/polym8040161

**Published:** 2016-04-22

**Authors:** Anne Linhardt, Michael König, Wolfgang Schöfberger, Oliver Brüggemann, Alexander K. Andrianov, Ian Teasdale

**Affiliations:** 1 Institute of Polymer Chemistry, Johannes Kepler University Linz (JKU), Altenberger Straße 69, A-4040 Linz, Austria; anne.linhardt@jku.at (A.L.); michael.koenig@jku.at (M.K.); oliver.brueggemann@jku.at (O.B.); 2 Institute of Organic Chemistry, Johannes Kepler University Linz (JKU), Altenberger Straße 69, A-4040 Linz, Austria; wolfgang.schoefberger@jku.at; 3 Institute for Bioscience and Biotechnology Research, University of Maryland, 9600 Gudelsky Drive, Rockville, MD 20850, USA; andria1@umd.edu

**Keywords:** Peptide-polymer hybrid, biodegradable polymer, peptide-polymer conjugate, polyphosphazene, polymer therapeutics, hydrolytic degradation, enzymatic degradation, imiquimod (R837)

## Abstract

A novel series of peptide based hybrid polymers designed to undergo enzymatic degradation is presented, via macrosubstitution of a polyphosphazene backbone with the tetrapeptide Gly-Phe-Leu-Gly. Further co-substitution of the hybrid polymers with hydrophilic polyalkylene oxide Jeffamine M-1000 leads to water soluble and biodegradable hybrid polymers. Detailed degradation studies, via ^31^P NMR spectroscopy, dynamic light scattering and field flow fractionation show the polymers degrade via a combination of enzymatic, as well as hydrolytic pathways. The peptide sequence was chosen due to its known property to undergo lysosomal degradation; hence, these degradable, water soluble polymers could be of significant interest for the use as polymer therapeutics. In this context, we investigated conjugation of the immune response modifier imiquimod to the polymers via the tetrapeptide and report the self-assembly behavior of the conjugate, as well as its enzymatically triggered drug release behavior.

## 1. Introduction

Polypeptides have gained importance for biomedical applications during the last decades due to their unique physical, chemical and biological properties [1,2]. For instance, poly(glutamic acid), poly(lysine) or poly(aspartate) have been heavily-investigated as polymer therapeutics, meeting most of the requirements including biocompatibility, biodegradability, high drug loading capacity and non-toxicity [1]. Moreover, peptide–polymer conjugates make up an interesting new class of polymeric materials combining the advantages of both peptides and synthetic polymers to generate hybrid materials with novel properties that cannot be realized with one of the components alone [2,3,4,5,6]. They take advantage of the flexibility of polymer synthesis and diverse peptide functionality and properties. Furthermore, responsiveness of peptides to external stimuli can be exploited to create smart materials that change structure, size or other properties, when desired. A further important aspect of polypeptides is their inherent capacity to adopt stable conformations and self-assemble into highly organized nanoscale structures [4]. Potential applications of peptide–polymer conjugates are in both biological and non-biological applications, but a vast majority of the work to date has focused on biomedical applications such as drug delivery [7,8,9], imaging [10], tissue engineering [11] or vaccines [12]. Peptide functionalization or grafting of synthetic polymers improves for example, degradation profiles [13,14], drug loading and release [8,15,16], cell adhesion [17] or controlled self-assembly to superstructures [18].

Herein, we present a hybrid approach combining inorganic polyphosphazenes decorated with oligopeptides. Poly(organo)phosphazenes are a versatile class of polymers with immense potential for application in nanomedicine [19,20,21]. Recent advances in polyphosphazene synthesis allow controlled molecular weights, narrow molecular weight distributions [22,23,24], controlled hydrodynamic volumes and high water solubility [25]. Furthermore, their properties can easily be tuned via facile post-polymerization modification to obtain polymer–drug conjugates with tailored characteristics. The most remarkable properties of poly(organo)phosphazenes, which distinguish them from many other known high molecular weight polymers, e.g., poly(ethylene glycol) PEG [26] and *N*-(2-hydroxypropyl)methacrylamide (HPMA) [27], used as drug delivery systems, is that their high synthetic flexibility and loading capacity is combined with an inherently hydrolytically degradable backbone (to non-toxic degradation products) [28,29], an essential feature in avoiding the deleterious effects associated with post-drug-release accumulation of high molecular weight macromolecules in the organism [30]. Thus, the development of fully biodegradable polymeric drug delivery systems with molecular weights above the renal clearance limit but with non-toxic degradation products with molecular weights under the renal clearance limit are of significant importance for drug delivery applications via parenteral administration [30,31]. In addition, controlled intracellular drug release is also required and can be obtained by incorporating stimuli sensitive linkers in between the polyphosphazene backbone and drug [32].

Herein, a series of hybrid polymers consisting of the enzymatic degradable tetrapeptide and the unique biodegradable backbone of poly(organo)phosphazenes in combination with hydrophilic, oligomers (Jeffamine M-1000) to insure water solubility and controlled hydrodynamic volumes are presented. Many different, selectively degradable peptide sequences are described in the literature [33,34], but this initial study focuses on the Gly-Phe-Leu-Gly (GFLG) sequence, with it being well-reported to be susceptible to cathepsin B catalyzed hydrolysis in the intracellular lysosomal compartment [16,33] due to its hydrophobic amino acid residues in P_2_ and P_3_ positions enabling an energetically favorable interaction with the active site of the lysosomal enzyme [15,35]. GLFG has been extensively studied as linker or spacer in combination with synthetic polymers like HPMA [8,13,14,33] or PEG [9] for drug and gene delivery applications to obtain lysosomal drug release or degradable drug carriers. In this context, we also investigated the covalent linkage of drugs to the degradable carriers, leading to both enzymatic controlled drug release and simultaneous initiation of the degradation of the polyphosphazene backbone, due to the peptide is bound directly to the polyphosphazene backbone. The immune response modifier imiquimod (R837), which shows great potential for cancer immunotherapy, is used as exemplary drug [36]. However, the carbonic acid chain end of the peptide offers the possibility to also attach other different drugs like doxorubicin [9], epirubicin [37], adriamycin [15] and cyclopamine [38]. The synthesis, characterization and self-assembly of the hybrid polymers are presented, as well as detailed degradation studies of the polymers and the drug conjugate.

## 2. Materials and Methods

All solvents were dried using standard laboratory procedures. Synthesis of polymers was carried out either in a glove box (MBRAUN) under argon or under nitrogen using standard Schlenk line techniques. The polyetheramine copolymer (PEO-PPO-NH_2_) with an ethylene oxide/propylene oxide ratio of 19/3 and a *M*_n_ of 1000 g mol^−1^, sold under the trade name Jeffamine M-1000, was donated by Huntsman Performance Products (Huntsman Holland B.V., Rotterdam, The Netherlands) and used as received. PCl_5_ was purified by sublimation and stored under argon. Triethylamine was distilled and dried over molecular sieves prior to use. Fmoc- amino acids, COMU and DIEA were purchased from Novabiochem (Merck, Darmstadt, Germany). TAEA was purchased from Acros Organics (Geel, Belgium), solvents were from VWR-Chemicals (Leuven, Belgium) and used without further purification. Other chemicals were purchased from Sigma Aldrich (Steinheim, Germany) or TCI chemicals (Zwijndrecht, Belgium).

^1^H-NMR spectroscopy was recorded on a Bruker 300 or 400 MHz spectrometer (Billerica, MA, USA) and referenced to the signal of internal CDCl_3_. ^31^P NMR spectra were recorded in decoupled mode on the same spectrometers at 121 or 162 MHz, using 85% phosphoric acid as an external standard. Gel permeation chromatography (GPC) was measured with a Viscothek GPCmax instrument (Malvern Istruments, Malvern, UK) equipped with a PFG column from PSS (Mainz, Germany; 300 mm × 8 mm, 5 µm particle size). The samples were eluted with DMF containing 10 mM LiBr at a flow rate of 0.75 mL/min at 60 °C. The molecular weights were estimated using a conventional calibration of the refractive index detector *versus* linear polystyrene standards. ATR-FTIR spectra were measured on a Perkin Elmer Spectrum 100 FTIR spectrometer (Waltham, MA, USA). A Malvern ZetaSizer Nano-ZS analyzer (Malvern Instruments, Malvern, UK) was used for dynamic light scattering (DLS) measurements. The 4 mW HeNe laser was set at λ = 633 nm with the detector angle at 173° for backscattering measurements. The measurements were carried out in buffer solutions (1 mg/mL) and all samples were filtered through a Millipore Millex-GV (Billerica, MA, USA) 0.22 µm PVDF filter and measured in a disposable polystyrene ultra-micro cuvette at 25 °C. UV–Vis spectra were carried out on a Perkin Elmer Lambda 25 UV/VIS spectrophotometer (Waltham, Massachusetts, USA). Field flow fractionation measurements were carried out on a Postnova AF2000 Ambient Temperature Asymmetric Flow FFF (Salt Lake City, UT, USA) equipped with an UV detector. A 1290 Infinity UHPLC from Agilent Technologies (Agilent, Vienna, Austria) equipped with a reversed-phase C18 silica-based chromatographic column (Rapid Resolution HD Eclipse Plus C18; 2.1 mm × 50 mm, particle size 1.8 µm) was used for kinetic studies of the drug release. The samples were eluted at a flow rate of 0.3 mL min^−1^ at room temperature with a mobile phase composition of 20% acetonitrile in water (*v*/*v*) containing 0.1% formic acid (*v*/*v*) in isocratic mode. UV detection was carried out at 254 nm. The amount of the released drug was estimated using a calibration curve for the free drug.

### 2.1. Tetra Peptide Synthesis (Gly-Phe-Leu-Gly-OtBu)

First peptide coupling: In a 100 mL round bottom flask 1.167 g (3.3 mmol) of Fmoc-Leu-OH were suspended in 30 mL EtOAc. Subsequently, 1.15 mL (6.6 mmol) DIEA and 1.413 g (3.3 mmol) COMU were added and the mixture was stirred for 3 min until a deep orange color was obtained. After the addition of 504 mg (3 mmol) of H-Gly-OtBu·HCl the reaction was stirred for 1 h and the solvent was removed *in vacuo*.

General procedure for Fmoc deprotection using TAEA: The crude residue from the precedent coupling was dissolved in 20 mL dichloromethane, 11.25 mL (75 mmol) of TAEA were added and the reaction was stirred for 30 min. The mixture was transferred to a separatory funnel containing 100 mL EtOAc and 50 mL brine, the funnel was shaken and the layers were separated. The organic phase was washed two times with 50 mL brine and two times with 50 mL pH 5.5 phosphate buffer, dried over anhydrous MgSO_4_, and evaporated.

General procedures for COMU coupling: In a 50 mL round bottom flask 3.3 mmol of Fmoc- amino acid were suspended in 30 mL EtOAc, followed by the addition of 1.15 mL (6.6 mmol) DIEA and 1.413 g (3.3 mmol) COMU. The resulting mixture was stirred for 2 min, added directly to the residue from the precedent deprotection and the resulting mixture was stirred for 1 h. After completion of the reaction, the solvent was removed on a rotary evaporator.

Modified work-up after final coupling step: After completion of the peptide coupling, the reaction solution was diluted with 100 mL EtOAc and washed with 1m HCl (2 × 50 mL), saturated NaHCO_3_ (2 × 50 mL) and brine (2 × 50 mL). The organic phase was dried over anhydrous MgSO_4_, filtered and the solvent was evaporated. The resulting residue was dissolved in CH_2_Cl_2_ and purified via DCVC [39]: SiO_2_, cyclohexane/EtOAc 50:0 to 0:50 in 5% increments. The product was further eluted by applying additional fractions of EtOAc if necessary. The protected tetrapeptide could be obtained as 1.4 g (2.09 mmol) of a colorless foam in 70% yield. ^1^H NMR (300 MHz, DMSO-d_6_, δ): 8.14–8.09 (m, 2H, –CO–NH–), 8.01 (d, 1H, J^3^(H,H) = 8.3 Hz, –CO–NH–), 7.88 (d, 2H, J^3^(H,H) = 7.7 Hz, Fluorenyl C^5^H and C^4^H), 7.69 (d, 2H, J^3^(H,H) = 7.3 Hz, Fluorenyl C^1^H and C^8^H), 7.41 (t, 2H, J^3^(H,H) = 7.3 Hz, Fluorenyl C^3^H and C^6^H), 7.32 (t, 2H, J^3^(H,H) = 7.7 Hz, Fluorenyl C^2^H and C^7^H), 7.22–7.14 (m, 5H, Phe Ar–H), 4.56–4.51 (m, 1H, FmocNH–CH_2_–), 4.35–4.18 (m, 4H, Fluorenyl C^9^H and Fmoc –CH_2_–O– and Leu –CH_2_–CH–NH–), 3.72–3.45 (m, 4H), 3.02 (dd, 1H, J^3^(H,H) = 13.8 Hz, J^4^(H,H) = 4.4 Hz, Phe –CH(H)–Ph), 2.80–2.73 (m, 1H, Phe –CH(H)–Ph), 1.62–1.56 (m, 1H, Leu –CH(CH_3_)_2_), 1.48 (t, 2H, J^3^(H,H) = 7.3 Hz), Leu –CH–CH_2_–CH(CH_3_)_2_), 1.39 (s, 9H, –COOC(CH_3_)_3_), 0.88–0.82 (m, 6H, Leu –CH(CH_3_)_2_) ppm.

Removal of the terminal Fmoc-group [40]: The purified tetrapeptide (1 g) was suspended in 19 mL MeCN and 1 mL DBU and 3.7 mL 1-dodecanethiol were added. The mixture was stirred for 1 h at room temperature followed by the addition of 15 mL *n*-heptane. The layers were separated and the MeCN phase was washed four times with 10 mL *n*-heptane, the solvent was evaporated and the residue dried under high vacuum. The crude product was directly used in the next step.

### 2.2. Synthesis of Cl_3_PNSiMe_3_

*N*-(trimethylsilyl)-trichlorophosphoranimine was synthesized similar to literature procedures [41]. LiN(SiMe_3_)_2_ (26 g, 155 mmol) was dissolved in 500 mL anhydrous diethylether under nitrogen at 0 °C and stirred for 30 min. Then, 13.59 mL PCl_3_ (155 mmol) were added dropwise at 0 °C. The solution was allowed to warm to room temperature and stirred for 1 hour. After cooling to 0 °C again, 12.56 mL SO_2_Cl_2_ (155 mmol) were added and the mixture was stirred for another hour at 0 °C. Afterwards, the reaction was filtered and the solvent removed under vacuum. The product was purified by vacuum distillation at 40–50 °C and 5 mbar to yield chlorophosphoranimine as colorless liquid. The product was stored under inert argon atmosphere at –35 °C. Yield: 14 g (40%), ^1^H-NMR (300 MHz, CDCl_3_, δ): 0.18 (d, 9H) ppm, ^31^P NMR (121 MHz, CDCl_3_, δ): −54.3 ppm.

### 2.3. Polymerisation Procedure

The polymer was synthesized via the living cationic polymerization of trichlorophosphoranimine [23] with PCl_5_. In the following, the procedure used for the synthesis of polymer **2** is described. Polymers **3** and **4** were synthesized accordingly, with varied ratio of peptide to Jeffamine M-1000. For polymer **5**, H-Gly-Jeffamine M-1000 was used as hydrophilic sidechain and H-Gly-Phe-Leu-Gly-imiquimod instead of H-Gly-Phe-Leu-Gly-OtBu. In the case of polymer **1**, no hydrophilic sidechain was used. The chlorine atoms were only substituted by H-Gly-Phe-Leu-Gly-OtBu. In the glove box, initiator PCl_5_ (13.0 mg, 0.0624 mmol) and monomer Cl_3_PNSiMe_3_ (0.350 g, 1.56 mmol) were dissolved in CH_2_Cl_2_ (5 mL) at room temperature. The solution was stirred for 12 h and the solvent removed under vacuum. The resulting polydichlorophosphazene (0.350 g, 1.56 mmol) was then dissolved in anhydrous THF in the glovebox. One equivalent of H-Gly-Phe-Leu-Gly-OtBu (0.700 g, 1.56 mmol) and NEt_3_ (0.16 g, 1.56 mmol) were then added to the polymer solution and allowed to react for 24 h. An excess of Jeffamine M-1000 (1.5 eq, 2.34 g, 2.34 mmol) was then added to the reaction mixture and allowed to react for further 24 h. The solvent was then removed under vacuum and the resulting polymer was purified by dialysis (12 kDa cut-off) for one week against EtOH. The solvent was removed and the polymer was dried under vacuum to give a waxy solid.

Polymer **1**:

Yield: 50%, ^1^H-NMR (300 MHz, CDCl_3_, δ): 0.86 (br, 6H), 1.45 (br, 9H) 2.26 (b, 1H), 3.63 (s, 82H), 7.18 (br 5H) ppm, ^31^P NMR (121 MHz, CDCl_3_, δ): 1 ppm; FTIR (solid): ν_max_ = 3282 (C–H), 2932 (N–H), 1740 (C(=O)–NH–), 1646 (C(=O)–OR), 1153 (C–O–C), 1034 (P=N).

Polymer **2**:

Yield: 0.5 g (22%), ^1^H-NMR (300 MHz, CDCl_3_, δ): 0.85 (br, 6H), 1.12 (br, 3H), 1.24 (br, 2H), 1.44 (br, 9H) 3.37 (s, 2H), 3.63 (m, 29H), 7.21 (br 5H) ppm, ^31^P NMR (121 MHz, CDCl_3_, δ): 0 ppm; FTIR (solid): ν_max_ = 3277 (C–H), 2869 (N–H), 1744 (C(=O)–NH–), 1660 (C(=O)–OR), 1097 (C–O–C), 1034 (P=N).

Polymer **3**:

Yield: 38%, ^1^H-NMR (300 MHz, CDCl_3_, δ): 0.88 (br, 4H), 1.13 (br, 3H), 1.26 (br, 3H), 1.45 (br, 9H) 3.38 (s, 2H), 3.66 (m, 39H), 7.20 (br 5H) ppm, ^31^P NMR (121 MHz, CDCl_3_, δ): 1 ppm; FTIR (solid): ν_max_ = 3276 (C–H), 2869 (N–H), 1744 (C(=O)–NH–), 1660 (C(=O)–OR), 1097 (C–O–C), 1034 (P=N).

Polymer **4**:

Yield: 38%, ^1^H-NMR (300 MHz, CDCl_3_, δ): 0.89 (br, 4H), 1.13 (br, 3H), 1.26 (br, 3H), 1.45 (br, 9H) 3.38 (s, 4H), 3.66 (m, 46H), 7.23 (br 5H) ppm, ^31^P NMR (121 MHz, CDCl_3_, δ): 0 ppm; FTIR (solid): ν_max_ = 3277 (C–H), 2869 (N–H), 1744 (C(=O)–NH–), 1660 (C(=O–OR), 1097 (C–O–C), 1038 (P=N).

Polymer **5**:

Yield: 30%, ^1^H-NMR (300 MHz, CDCl_3_, δ): 0.86 (br, 1H), 1.13 (br, 3H), 1.25 (br, 2H) 3.38 (s, 2H), 3.65 (s, 23,78H) ppm, ^31^P NMR (121 MHz, CDCl_3_, δ): 0 ppm; FTIR (solid): ν_max_ = 3253 (C–H), 2867,13 (N–H), 1740 (C(=O)–NH–), 1108 (C–O–C), 1034 (P=N).

### 2.4. Gly-Phe-Leu-Gly-Imiquimod

Firstly, Fmoc-Gly-Phe-Leu-Gly was stirred overnight in TFA to remove the *tert*-butyl protective group. TFA was removed under vacuum and CH_2_Cl_2_ was added to the product and evaporated three times to obtain a white powder. Then, 161 mg imiquimod (0.67 mmol) were dissolved in DMF containing 0.2 mL trimethylamine (1.34 mmol) by heating to 50 °C in DMF for 20 min. To the reaction mixture, 0.57 g EDCI (3 mmol) and 413 mg Fmco-Gly-Phe-Leu-Gly-OH (0.67 mmol) were added and left overnight. The reaction progression was indicated by TLC (EtOAc:cyclohexane, 1:1). The reaction was then diluted in EtOAc and washed two times with NaHCO_3_, two times with saturated NaCl and dried over MgSO_4_. The solvent was removed and the product was dried under vacuum. Yield: 0.38 g (68%).

### 2.5. Gly-Jeffamine M-1000

Gly-Jeffamine was synthesized similar to literature [29]. The BOC-protected amino acid BOC-Gly-OH (1.3 g, 7.5 mmol), *N*-hydroxysuccinimide (0.86 g, 7.5 mmol) and N,N´-dimethylaminopyridine (0.09 g, 7.5 mmol) were dissolved in 80 mL CH_2_Cl_2_ and cooled to 0 °C. Separately, 1.55 g *N*,*N*´-dicyclohexylcarbodiimide (7.50 mmol) were dissolved in 15 mL CH_2_Cl_2_ and transferred in to the cooled reaction mixture. The mixture was stirred overnight at room temperature. The formed precipitate was removed by filtration and the filtrate was added to a solution of 7.5 g Jeffamine M-1000 (7.5 mmol) in CH_2_Cl_2_ and stirred for two days at room temperature. The reaction was extracted two times with 10% ammonium chloride, two times with 5% NaHCO_3_, two times with saturated NaCl and dried over MgSO_4_. The solvent was removed and the product dried under vacuum to yield a white wax-like product. Yield 7.2 g (88%), ^1^H-NMR (300 MHz, CDCl_3_, δ): 1.39 (s, 9H), 3.31 (s, 3H), 3.58 (m, 82H) ppm.

### 2.6. Hydrolytic Degradation Study–Field Flow Fractionation

Polymer **2** (5 mg/mL) was incubated in pH 2, pH 5 and pH 7.4 citrate/phosphate buffer (0.1 M) at 37 °C during the time of analysis. Aliquots (50 µL) of the samples were taken after certain time intervals and measured via field flow fractionation.

### 2.7. Hydrolytic Degradation Study—Dynamic Light Scattering

Polymer **5** (1 mg/mL) was incubated in pH 2, pH 5 and pH 7.4 citrate/phosphate buffer (0.1 M) at 37 °C during the time of analysis in disposable polystyrene ultra-micro cuvettes and measured after different times at 25 °C.

### 2.8. Enzymatic and Hydrolytic Degradation Study–NMR

For ^31^P NMR degradation studies, 10 mg papain (13 units/mg) were activated with 0.01 M L-cysteine in 0.9 mL citrate buffer (0.1 M, pH 6) and added to 10 mg polymer. Separately, 10 mg polymer were dissolved in 0.9 mL of the same solution but without papain. Moreover, 10 mg papain were mixed with 0.9 mL buffer containing 0.01 M of the inhibitor cystamine. Although phosphate buffer is reported to be the best choice for papain [42], citrate buffer had to be used to avoid buffer related signals in the ^31^P NMR spectra. Immediately when the polymer was dissolved, 0.1 M D_2_O was added to the solutions and the sample incubated at 37 °C. The changes of the phosphorus signals were monitored over a time period of 28 days. Between each measurement the samples were stored at 37 °C.

### 2.9. Degradation—Phosphate Determination

The polymers (1.3 mg/mL) were incubated in pH 5 sodium acetate buffer (0.1 M) containing 0.01 M L-cysteine and 0.057 mM Papain (3 units/mg), at 37 °C during the time of analysis. Hydrolytic degradation was measured under the same conditions but without papain. Aliquots (0.4 mL) of the samples were taken after certain time intervals and tested for the presence of inorganic phosphate using 1 mL of a reagent solution containing 5 mL of 0.4% ammonium molybdate, 5 mL of 0.7% ascorbic acid, 12.5 mL of 0.2 M sulfuric acid, and 2.5 mL of 0.018% potassium antimonyl tartrate [43]. UV–Vis analysis of the mixtures was performed at 885 nm after 15 min incubation. The concentration of phosphate was calculated using a calibration curve measured with potassium dihydrogen phosphate and compared to the theoretical phosphate amount that can be released from the polymer backbone. Although phosphate buffer is reported to be the best choice for papain [42], acetate buffer had to be used to avoid buffer related signals in the UV–Vis measurements.

### 2.10. Drug Release—HPLC Measurements

The release of imiquimod from the polymer **5** was analyzed by HPLC. Papain (10 mg, 3 units/mg) was dissolved in 1 mL 0.1 M citrate buffer containing 0.01M L-cysteine and stirred for 5 minutes to activate the enzyme and subsequently added to 10 mg of polymer **5**. The amount of released drug was then investigated by HPLC measurements after certain periods of times and the samples were stored at 37 °C between each measurement. UV detection was carried out at 254 nm and the amount of the released drug was estimated using a calibration curve for the free drug measured under the same conditions. For comparison, the drug release was also studied without papain to investigate the hydrolytic drug release. In this case, polymer **5** was dissolved in 1 mL 0.1 M citrate buffer containing 0.01 M L-cysteine and stored and measured like the papain containing sample.

## 3. Results

### 3.1. Synthesis

**Figure 1:**
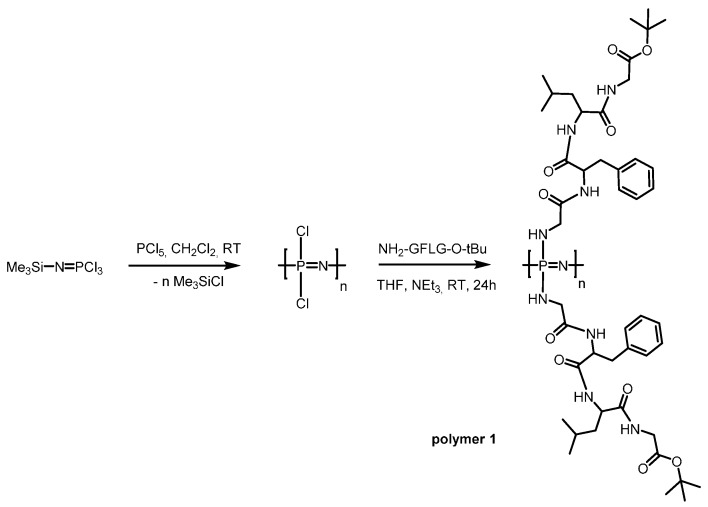
Living polymerisation of poly(dichlorophosphazene) and macromolecular substitution of the chlorine atoms by Gly-Phe-Leu-Gly-OtBu.

**Figure 2:**
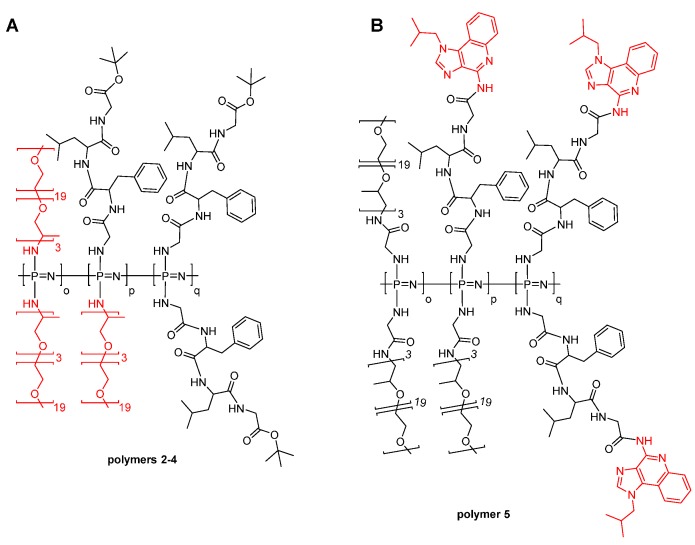
(**A**) Poly(organo)phosphazenes **2**–**4** with the tetrapeptide linker GFLG and Jeffamine M-1000 coupled to the polyphosphazene backbone. (**B**) Poly(organo)phosphazene **5** loaded with imiquimod via the tetrapeptide linker GFLG and Jeffamine M-1000 coupled via a glycine spacer to the polyphosphazene backbone. The combinations of the two different side chains are statistically distributed.

Poly(dichloro)phosphazene [Cl_2_P=N]_n_ with approximately 50 repeat units was synthesized via the room temperature, living cationic polymerization of trichlorophosphoranimine [23,25]. This reaction was followed by the macromolecular substitution of the chlorine atoms with separately prepared H-Gly-Phe-Leu-Gly-OtBu to obtain peptide based poly(organo)phosphazene (Figure 1, polymer **1**). ^31^P NMR, ^1^H-NMR spectroscopy and GPC were used to confirm successful preparation of the hybrid polymer (Table 1 and Figures S1 and S2). With two tetrapeptide groups per repeat unit, the resulting hybrid polymer consists mostly of peptide (95 wt %), and thus, the chemical and solution characteristics are peptide dominated for the hybrid polymer. Due to the hydrophobicity of the peptide sequence, the resulting polymer had limited aqueous solubility; thus, a further series of polymers were prepared using Jeffamine M-1000 as a co-substituent in various ratios (polymers **2**–**4**, Figure 2A) to obtain hybrid polymers with excellent water solubility.

The relative ratio of the tetrapeptide and the Jeffamine side chains was calculated by ^1^H-NMR spectroscopy, through integration of the OCH_3_ Jeffamine end-group protons (s, 3H) at 3.37 ppm *versus* the *tert*-butyl-group (s, 9H) of the protected tetrapeptide sidechain at 1.44 ppm (Table 1). Furthermore, all polymers were characterized with ^31^P NMR spectroscopy. No peaks were resolved from P-Cl units, thus suggesting substitution of all chlorine atoms in the polyphosphazene backbone (Figure S2A,B).

A common tactic in polymer therapeutics involves the coupling of amino-functionalized drugs to the C-terminus of the Gly-Phe-Leu-Gly sequence, which can facilitate intracellular lysosomal drug release [9,15,37,38]. Thus, in this work, we coupled the immune response modifier imiquimod to Fmoc-Gly-Phe-Leu-Gly-OH via its aromatic NH_2_. After Fmoc-deprotection, the resulting conjugate H-Gly-Phe-Leu-Gly-imiquimod was added as co-substituent alongside H-Gly-Jeffamine M-1000 to –[Cl_2_P=N]–, to obtain polymer **5** (Figure 2B). The amount of imiquimod attached to polymer **5** via the tetrapeptide linker was calculated using the UV-Vis absorbance at 246 nm (Figure S3) and gave a drug loading of 2.4 wt % of the conjugate (Table 1).

### 3.2. Self-Assembly

**Figure 3:**
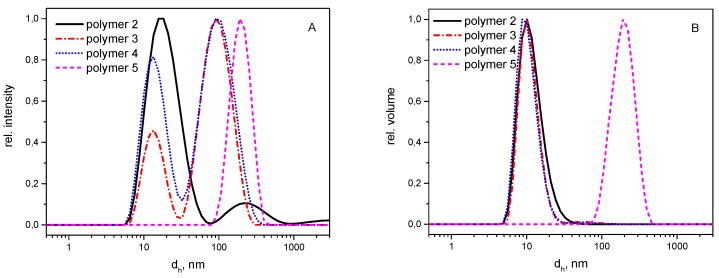
Molecular size distribution by intensity (**A**) and volume (**B**) as detected by dynamic light scattering for polymers **2**–**5** in phosphate buffer at pH 7.4 (polymer concentration 1 mg/mL, d_h_—hydrodynamic diameter).

**Figure 4:**
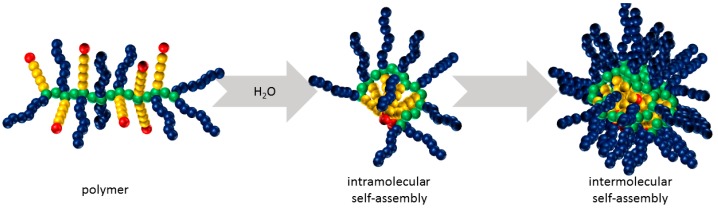
Intra- and intermolecular self-assembly of poly(organo)phosphazenes with amphiphilic character in aqueous solution.

The hydrodynamic volumes of the hybrid polymer series was then investigated by dynamic light scattering (DLS, Figure 3) to determine the hydrodynamic diameter in aqueous solutions, a factor known to have considerable impact on plasma circulation time, cellular uptake and biodistribution of the polymers. Figure 3A shows the size distribution by intensity and Figure 3B by volume of polymers **2**–**5**. The intensity distribution of polymers **2**–**4** show a bimodal distribution, hinting at some self-assembly of the polymers. Such behavior has been reported previously for similar amphiphilic co-substituted polyphosphazenes [44,45,46,47,48,49,50,51]. Micellar-like superstructures with the hydrophobic side groups agglomerated in the core can be formed despite them being essentially random copolymers, presumably due to the high flexibility of the backbone, allowing for folding and agglomeration of the hydrophobic sections (Figure 4).

Interestingly, the distribution was observed to be monomodal in the region of 200 nm for polymer **5**, loaded with the hydrophobic drug, suggesting this exists mostly in its agglomerated form for 1 wt % solutions. Since larger particles show higher intensity, a closer inspection of the size distribution by volume was also carried out (Figure 3B). In summary, the DLS investigations show that although all polymers tend to form aggregates due to their amphiphilic character resulting from the combination of hydrophilic Jeffamine sidechains with the hydrophobic tetrapeptide, only in the case of polymer **5** are the formed aggregates the dominating species present in the sample as a result of the increased hydrophobicity imparted by the hydrophobic drug.

### 3.3. Hydrolytic Degradation

**Figure 5:**
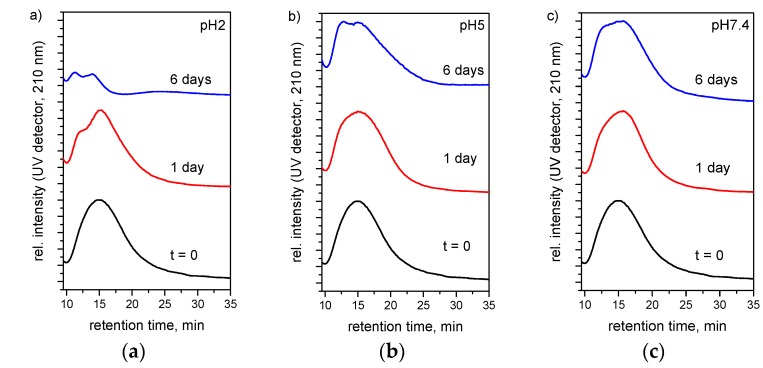
Normalized FFF analysis illustrating the degradation of polymer **2** at 37 °C, in an aqueous solution at pH: 2 (**a**); 5 (**b**); and 7.4 (**c**). Broadening and decrease in intensity and a shift to earlier elution time of the polymer peak are observed.

Degradation studies of polymer **2** measured by field flow fractionation at 37 °C, pH 2, 5 and 7.4 showed that the polymers are stable over a short period of time in an aqueous environment but degrade significantly to small molecules under simulated physiological conditions (pH 5 and 7.4) with a broadening and a shift to earlier retention times of the polymer peak being observed (Figure 5). A more rapid degradation occurred under enhanced (pH 2) conditions (Figure 5a), with the entire polymer being observed to degrade within two weeks (Figure S4), in accordance with previous reports into amino acid substituted poly(organo)phosphazenes [29].

**Figure 6:**
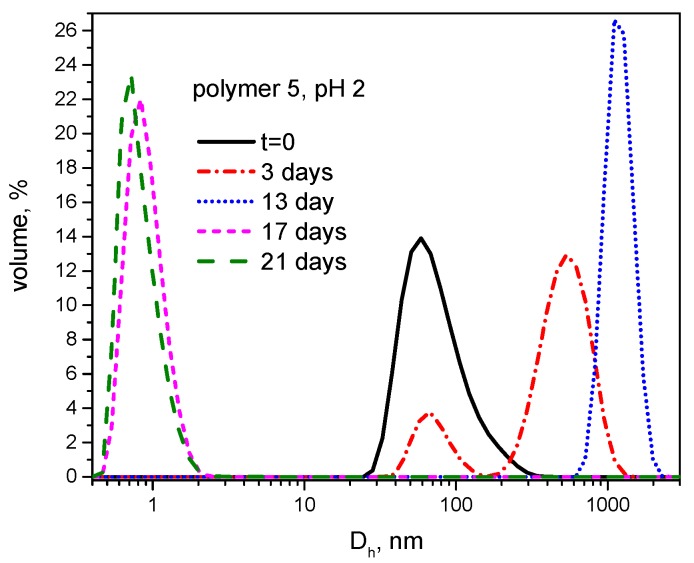
Size distribution by volume measured by dynamic light scattering for polymer **5** in citrate/phosphate buffer at pH 2 at various time points (polymer concentration—1 mg/mL, D_h_—hydrodynamic diameter).

Moreover, the hydrolytic degradation of polymer **5** was followed by DLS under acidic conditions (pH 2) to examine the behavior of supramolecular structures during the degradation (Figure 6). Interestingly, degradation was characterized by an initial increase in the size of aggregates, followed by the breakdown of particulates into soluble products in a nanometer size range. This phenomenon can potentially indicate differences in the degradation rates in hydrophilic and hydrophobic domains of the supramolecular assemblies. It is possible that a faster scission rate of polyether side groups can lead to a temporary “hydrophobization” of the polymer resulting in the enhanced aggregation. After 17 days, only small molecules, presumably the M-1000 side chains, could be detected (Figure 6).

### 3.4. Enzymatic Degradation

**Figure 7:**
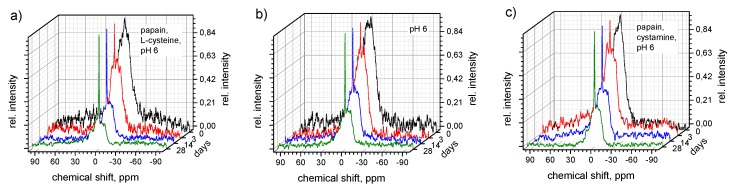
Enzymatic degradation of polymer **2** followed by ^31^P NMR spectroscopy over 28 days in citrate buffer (pH6) containing L-cysteine and papain (**a**); hydrolytic degradation of polymer **2** in the same buffer system without papain (**b**); and with papain and cystamine as inhibitor (**c**). All samples were stored at 37 °C.

Further to the hydrolytic degradation, the degradation of the hybrid polymers in the presence of the enzyme was also investigated. In these studies, a combination of papain with L-cysteine as an activator was employed to model a phosphate containing cathepsin B—an important lysosomal protease. This allowed using ^31^P-NMR spectroscopy and photometric determination of phosphates for monitoring degradation products. ^31^P-NMR spectra for polymer **2** at various degradation time points are shown in Figure 7. A sharp peak at around 0 ppm appeared in all ^31^P-NMR spectra after one day of incubation and increased over time, whereas the broad signal associated with the polymer decreased. Solutions that did not contain papain displayed a pronounced, but slower hydrolytic degradation under the same conditions (Figure 7b). Similar results were observed in the presence of the papain inhibitor cystamine (Figure 7c). These results appear to suggest the presence of a contribution from enzymatic degradation, as well as the clear hydrolytic response.

**Figure 8:**
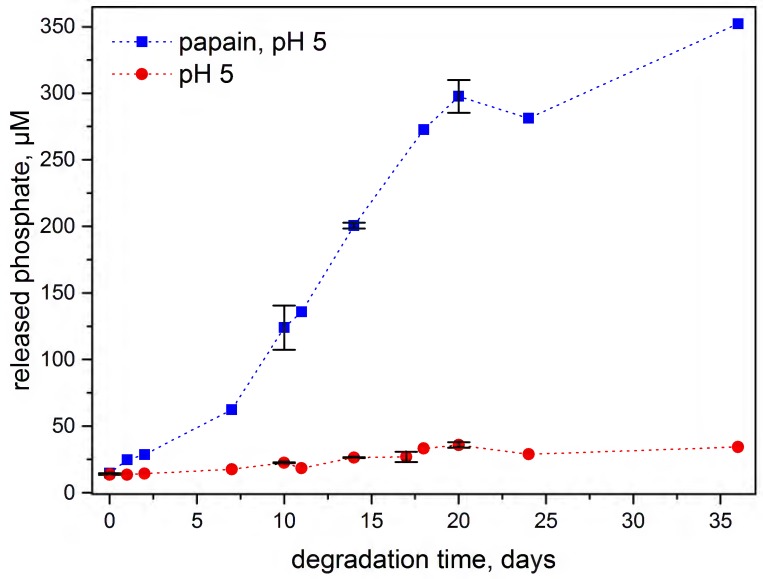
Phosphate determination of polymer **2** quantitatively determined by UV–Vis analysis to show the degradation profile of the polymer in aqueous conditions at pH 5 (■) and with papain at pH 5 (●)at 37 °C.

Furthermore, formation of phosphate could also be tracked by photometric molybdate assay [29]. The release of phosphate from polymer **2** was investigated at 37 °C, pH 5 (in acetate buffer due to the incompatibility of citrate buffer for the molybdate assay). Considerably higher degradation rates were observed for the polymer incubated with the l-cysteine activated papain than in its absence, suggesting a significant contribution from enzymatic degradation of the polymer (Figure 8).

### 3.5. Degradation Mechanism

**Figure 9:**
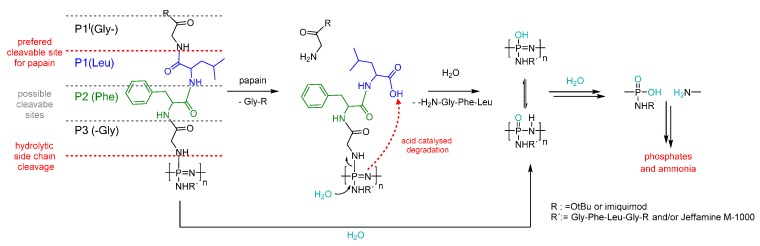
Preferential cleavable site of papain and proposed hydrolytic and enzyme initiated degradation mechanism of GFLG-peptide based poly(organo)phosphazenes.

As previously discussed, the hybrid polymers show a hydrolytic labile behavior, due to glycine bound directly to the polyphosphazene backbone, which could be indicated by the ^31^P NMR spectra as well as photometric phosphate determination. The degradation rate was clearly accelerated by the addition of the enzyme papain, which has similar specificity as the lysosomal enzyme cathepsin B [31,35], suggesting the involvement of an enzymatic degradation route. Papain has the preference for phenylalanine in position P2 (Schechter and Berger nomenclature) of the substrate [38]. Consequently, a cleavage between leucine and glycine-OtBu or glycine-imiquimod is expected (Figure 9). Despite the peptide preferential cleavage site not being directly at the polyphosphazene backbone, the degradation rate was nevertheless observed to be significantly accelerated upon the addition of papain. This observation is explained with an acid-catalyzed mechanism (Figure 9), whereby after enzymatic cleavage of a peptide bond, free carboxyl groups are formed, which would be expected to promote the backbone degradation. This carboxylic acid-catalyzed degradation mechanism has already been proposed for poly(organo)phosphazenes substituted with amino acid esters [44,45]. Moreover, it is to be expected that the entire peptide subsequently disintegrates into its corresponding amino acids in presence of enzymes like papain [16,38], thus leading directly to the hydroxyphosphazene degradation intermediate. Thus, it is proposed that, under lysosomal conditions, two different peptide cleavage mechanisms may occur, both of which would promote backbone degradation.

### 3.6. Drug Release

**Figure 10:**
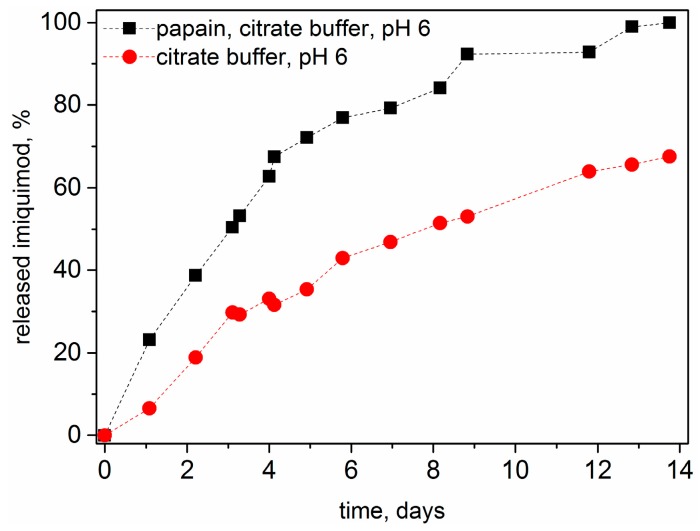
Hydrolytic release of imiquimod from polymer **5** at 37 °C in acidic environment (citrate buffer, pH 6) (●), and enzymatic release of imiquimod from polymer **5** at 37 °C in the same buffer with L-cysteine activated papain (■). The amount of the released drug was estimated using a calibration curve for the free drug.

The release of imiquimod from the polymer **5** was analyzed by HPLC and the amount of the released drug was estimated using a calibration curve for the free drug. The samples were stored at 37 °C between each measurement and the investigations were carried out in citrate buffer at pH 6 and with papain in the same buffer system. Within a period of 14 days, 100% release from the polymer–drug conjugate could be observed for the sample exposed to papain and only 65% for the conjugate stored at pH 6 without papain (Figure 10). This observation, suggests that, as expected hydrolytic and enzymatic drug release take place simultaneously. According to the supposed mechanism, Gly-imiquimod is preferentially released by papain but according to other comparable published data from authors using similar GFLG based macromolecular drug delivery systems, Gly-drug degrades eventually to glycine and free drug [16,38]. It is assumed that the bond between glycine and drug is also a secondary cleavage site for papain. If used, as proposed, in polymer therapeutics *in vivo*, the lysosomal cathepsin B would be expected to preferentially cleave the Gly-imiquimod bond [16,38].

## 4. Conclusions

A series of novel peptide hybrid polymers are reported via the grafting of the tetrapeptide sequence Gly-Phe-Leu-Gly onto a polyphosphazene backbone with successful synthesis being confirmed by GPC, ^31^P and ^1^H NMR spectroscopy. The polymers showed excellent solubility in water upon co-substitution with hydrophilic side chain Jeffamine M-1000. Degradation studies showed the polymers degraded via both enzymatic, as well as hydrolytic pathways, with the degradation rates significantly enhanced in the presence of papain. The degradation products include phosphate and ammonia, as well as the respective amino acids and Jeffamine M-1000 oligomers. A suggested application of such materials is polymer therapeutics due to their showing sufficient stability under physiological conditions to be used as drug carriers delivering drugs via bloodstream, while subsequently disintegrating into low-molecular weight, non-toxic degradation products capable of undergoing renal clearance. This, combined with the observed spontaneous self-assembly upon conjugation of the hydrophobic drug imiquimod, renders the presented degradable polymers potentially interesting for drug delivery applications.

## Figures and Tables

**Table 1:** Structural data for polymers **1**–**5**.

Polymer	GFLG-OtBu, % ^a^	Jeffamine M-1000, % ^a^	Imiquimod loading, wt % ^b^	*M*_w_/*M*_n_ (GPC) ^c^	*M*_n_ (GPC), kDa ^c^	D_h_ (DLS), nm ^d^
**1**	100	0	0	1.3	6.84	– ^f^
**2**	84	16	0	1.9	5.13	20,1 ± 1.6
**3**	57	43	0	1.9	4.05	14.2 ± 0.58
**4**	47	53	0	1.6	5.58	14.9 ± 0.58
**5**	– ^e^	– ^e^	2.4	1.6	6.20	201.6± 17.32

^a^ percentage of total substituents calculated from ^1^H NMR measurements; ^b^ weight percent of total conjugate calculated from UV–Vis measurements; ^c^ GPC analysis in DMF (10 mM LiBr), underestimated due to calibration with linear polystyrene standards; ^d^ peak mean from DLS size distribution by intensity in phosphate buffer (pH 7.4); ^e^ calculation not possible due to overlapping NMR-signals of side chains and imiquimod; ^f^ not soluble in water.

## Supplementary Materials

The following are available online at ww.mdpi.com/2073-4360/8/4/161/s1, **Figure S1:** GPC chromatographs of polymer **1–5**. All polymers elute at similar retention volumes indicating similar molecular weights, **Figure S2:** A: 31P NMR spectrum of polymer **1**–**5**. A broad peak at about 0 ppm indicates substitution of the chlorine atoms at polyphosphazene backbone and the absence of degradation products. B: 31P NMR spectrum of the monomer (*N*-(trimethylsilyl)-trichlorophosphoranimine), the precursor polymer (poly(dichlorophosphazene) and polymer **2** to show monomer conversion and macromolecular substitution of the chlorine atoms at the polyphosphazene backbone., **Figure S3**: UV–Vis spectra in acetonitrile of polymer **5** loaded with 2.4 wt % imiquimod (black), of imiquimod (red) and of Gly-Phe-Leu-Gly-imiquimod (blue), **Figure S4**: Normalized FFF analysis illustrating the degradation of polymer **2** at 37 °C in an aqueous solution at pH 2. Broadening and decrease in intensity and a shift to earlier retention time of the polymer peak are observed, **Figure S5**: Hydrolytic and enzymatic release of imiquimod from polymer **5** during 3.5 days shown in two different studies to confirm reproducibility, **Figure S6**: ATR-FTIR spectra of polymer **1**–**5**, **Figure S7**: Enzymatic degradation of polymer **2** followed by ^31^P NMR spectroscopy after 14 days in citrate buffer (pH 6) containing L-cysteine and papain (black), hydrolytic degradation of polymer **2** in the same buffer system without papain (red) , with papain and cystamine as inhibitor (blue). All samples were stored at 37 °C, **Figure S8**: Enzymatic degradation of polymer **2** followed by ^31^P NMR spectroscopy of two different samples a-1 and a-2) under the same conditions (28 days in citrate buffer (pH 6) containing l-cysteine and papain). All samples were stored at 37 °C.

## Abbreviations

The following abbreviations are used in this manuscript:

COMU	1-Cyano-2-ethoxy-2-oxoethylidenaminooxy)dimethylamino-morpholino-carbenium hexafluorophosphate
DBU	1,8-Diazabicyclo[5.4.0]undec-7-ene
DIEA	*N*,*N*-Diisopropylethylamine
DLS	dynamic light scattering
DMF	Dimethylformamide
EDCI	1-Ethyl-3-(3-dimethylaminopropyl)carbodiimide
FFF	field flow fractionation
Fmoc	9-Fluorenylmethoxycarbonyl
PEG	polyethylene glycol
GFLG	Glycine-Phenylalanine-Leucine-Glycine
Gly	Glycine
GPC	gel permeation chromatography
HPLC	high performance liquid chromatography
HPMA	*N*-(2-hydroxypropyl)methacrylamide
Leu	Leucine
NMR	Nuclear magnetic resonance
PEG	polyethylene glycol
Phe	Phenylalanine
TAEA	Tris(2-aminoethyl)amine
TFA	Trifluoroacetic acid
THF	Tetrahydrofuran
UV–Vis	Ultraviolet–Visible
