# Supplementary material for: Biodegradable Polyphosphazene Based Peptide-Polymer Hybrids
Source: Polymers (Basel). 2016 Apr 22;8(4):161. doi: 10.3390/polym8040161 (PMC6432119; doi:10.3390/polym8040161)
Supplement: Supplementary file 1 [file polymers-08-00161-s001.pdf]

Supplementary Materials: Biodegradable Polyphosphazene Based Peptide-Polymer Hybrids

Anne Linhardt, Michael König, Wolfgang Schöfberger, Oliver Brüggemann, Alexander K. Andrianov and Ian Teasdale

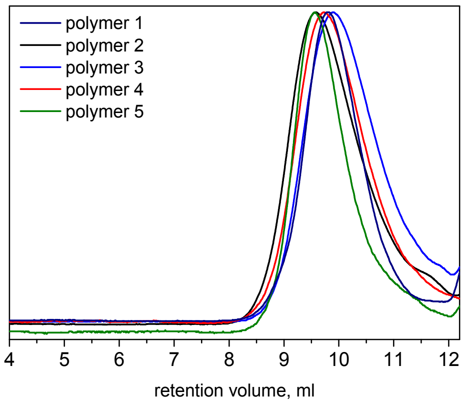

Figure S1. GPC chromatographs of polymer **1–5**. All polymers elute at similar retention volumes indicating similar molecular weights.

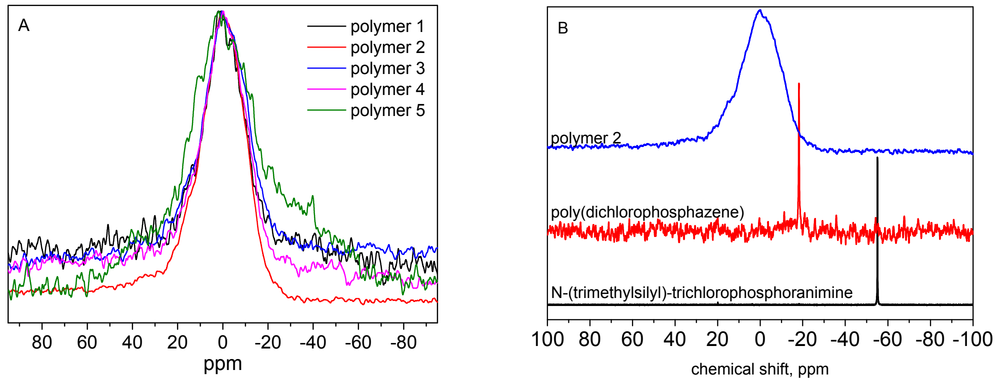

Figure S2. (**A**) ^{31}P NMR spectrum of polymer **1–5**. A broad peak at about 0 ppm indicates complete substitution of the chlorine atoms at polyphosphazene backbone and the absence of degradation products. (**B**) ^{31}P NMR spectrum of the monomer (*N*-(trimethylsilyl)-trichlorophosphoranimine), the precursor polymer (poly(dichlorophosphazene) and polymer 2 to show complete monomer conversion and macromolecular substitution of the chlorine atoms at the polyphosphazene backbone.

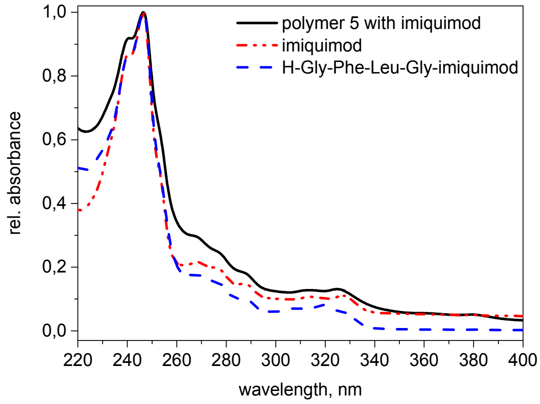

Figure S3. UV–Vis spectra in acetonitrile of polymer **5** loaded with 2.4 wt % imiquimod (black), of imiquimod (red) and of Gly-Phe-Leu-Gly-imiquimod (blue).

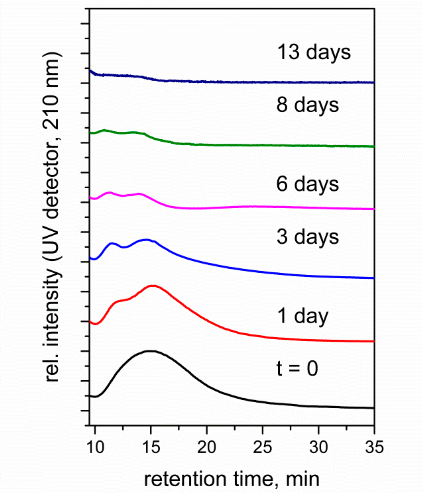

Figure S4. Normalized FFF analysis illustrating the degradation of polymer **2** at 37 °C in an aqueous solution at pH 2. Broadening and decrease in intensity and a shift to earlier retention time of the polymer peak are observed.

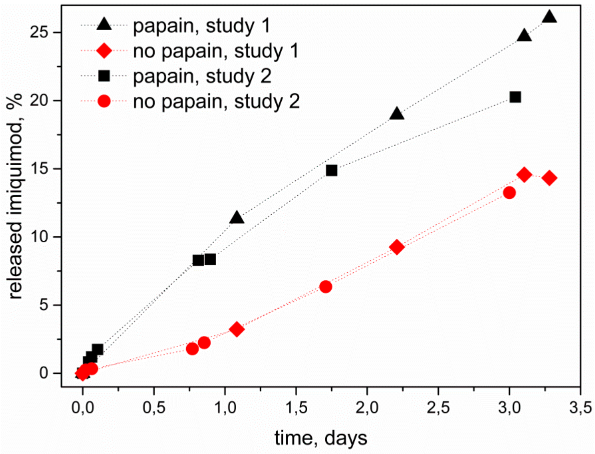

Figure S5. Hydrolytic and enzymatic release of imiquimod from polymer **5** during 3.5 days shown in two different studies to confirm reproducibility.

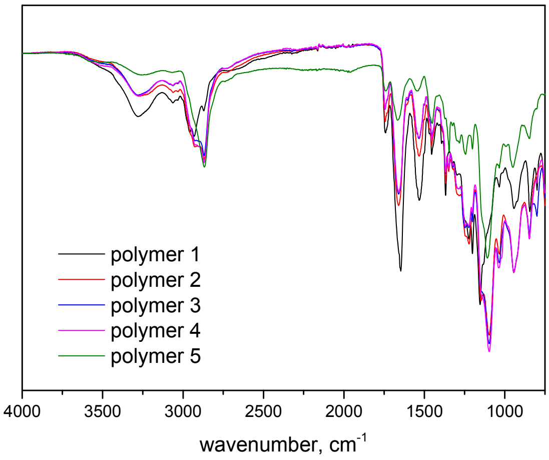

Figure S6. ATR-FTIR spectra of polymer **1–5**.

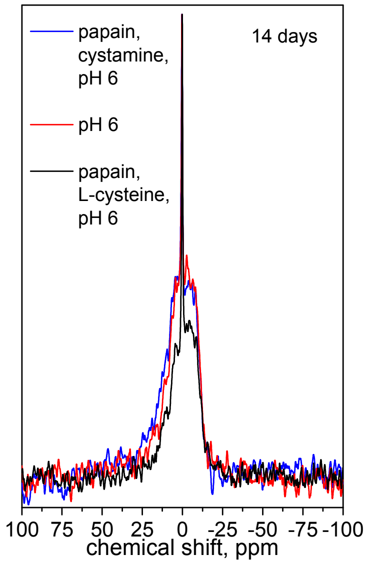

Figure S7. Enzymatic degradation of polymer 2 followed by ^{31}P NMR spectroscopy after 14 days in citrate buffer (pH 6) containing L-cysteine and papain (black), hydrolytic degradation of polymer **2** in the same buffer system without papain (red), with papain and cystamine as inhibitor (blue). All samples were stored at 37 °C.

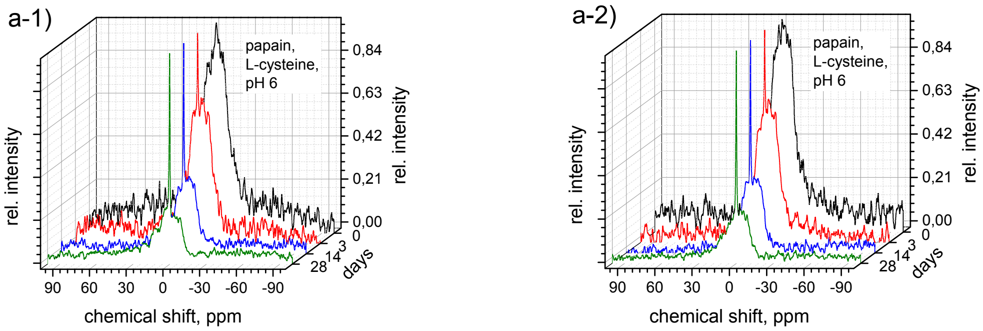

Figure S8. Enzymatic degradation of polymer **2** followed by ^{31}P NMR spectroscopy of two different samples a-1 and a-2) under the same conditions (28 days in citrate buffer (pH 6) containing L-cysteine and papain). All samples were stored at 37 °C.

© 2016 by the authors; licensee MDPI, Basel, Switzerland. This article is an open access article distributed under the terms and conditions of the Creative Commons Attribution (CC-BY) license (http://creativecommons.org/licenses/by/4.0/).
